# Low-Incidence, High-Consequence Pathogens

**DOI:** 10.3201/eid2002.131748

**Published:** 2014-02

**Authors:** Ermias D. Belay, Stephan S. Monroe

**Affiliations:** Centers for Disease Control and Prevention, Atlanta, Georgia, USA

**Keywords:** high-consequence pathogens, high mortality, anthrax, orthopoxviruses, hemorrhagic fevers, filoviruses, monkeypox, pathology, infectious disease pathology, viruses, bacteria, zoonoses

Infectious diseases that have epidemic or pandemic potential and spread rapidly through a population within a short time are an ongoing public health concern in industrialized and developing countries. Frequent exposure to infectious sources (e.g., food, infected animals, and vectors) or a high rate of person-to-person spread facilitates spread of these diseases. Foodborne illnesses and seasonal influenza are notable examples. These diseases typically are associated with high rates of illness and substantial societal and economic cost but relatively low rates of death in otherwise healthy persons. Other infectious diseases, in contrast, may occur infrequently but are associated with high rates of death. The low incidence of some of these diseases reflects effective public health prevention measures, such as vaccinations. For a select group of zoonotic infectious diseases with high death rates, the low incidence reflects infrequent spillover from an animal reservoir into humans. Often, humans represent a dead-end host for these pathogens, and person-to-person transmission is rare if appropriate infection control practices are followed. Many of the pathogens highlighted in the current issue of Emerging Infectious Diseases can collectively be described as low-incidence, high-consequence pathogens. Selected diseases caused by these pathogens are described below.

## Examples of High-Consequence Pathogens

### Rabies

Rabies, one of the oldest known infectious diseases, is nearly 100% fatal and continues to cause tens of thousands of human deaths globally (*1*). Canine rabies has been eliminated in North America and many South American and European countries, but it is still the source of most human rabies cases in other areas, primarily in many African and Asian countries (*2*,*3*). Urbanization and lack of aggressive rabies elimination programs may have contributed to resurgence of canine rabies–associated human deaths in several provinces in China (*4*,*5*). In the United States, the number of human deaths from rabies has declined to an average of 3 cases per year during the last several decades (*1*). Apart from a few imported canine rabies cases, most human cases in the United States resulted primarily from bat rabies virus variants. Nonetheless, suspected or confirmed human exposures in the United States result in tens of thousands of postexposure prophylaxis regimens every year (*6*).

### Smallpox

Despite the eradication of smallpox in 1980, concerns about intentional or accidental release of variola virus and its potential for severe disease and high rates of death (average 30%) have fueled research into the development of new diagnostic tests, therapies, and vaccines. Recent advances in biosynthetic technologies risking possible reconstitution of the virus have heightened these concerns. To bolster preparedness efforts, some countries have procured or retained smallpox vaccine supplies in their national stockpiles. Detection in the 1970s of a related orthopoxvirus that causes monkeypox (*7*), a similar but milder illness in humans that can be fatal in up to 10% of patients, raised concerns that this virus may replace the ecologic and immunologic niche created by the eradication of smallpox (*8*–*10*). Waning herd immunity after cessation of smallpox vaccination, which appears to cross-protect against monkeypox, might have facilitated spread of the virus in areas to which it is endemic (*10*). Ecologic factors, including changes in the environment and agent reservoirs, also might have contributed to changes in the incidence of monkeypox (*11*). However, in humans, monkeypox virus is less virulent and less transmissible than variola virus (*9*). Monkeypox can spread from person to person after prolonged contact with a patient or indirectly through exposure to body fluids or fomites contaminated with the virus (*12*). Monkeypox occurs endemically and in occasional outbreaks in central and western Africa, where the presumed natural reservoirs of the virus exist (*9*,*10*). The 2003 monkeypox outbreak in the United States clearly illustrated the potential for monkeypox virus or other zoonotic viruses to be transported great distances and spread quickly among immunologically naive populations (*9*).

### Hemorrhagic Fever Diseases

Hemorrhagic fever disease can be caused by several families of viruses, including arenaviruses, bunyaviruses, filoviruses, and flaviviruses. Filoviruses, which comprise 4 Ebola viruses pathogenic to humans and 1 Marburg virus species, have caused multiple outbreaks of hemorrhagic fever primarily in central and eastern Africa (*13*,*14*). Since 1976, 10 large filovirus outbreaks involving >100 persons have been documented from the Congo Basin, Gabon, Sudan, and Uganda (*15*). During these outbreaks, transmission chains resulted from direct person-to-person spread in households and nosocomial transmissions through contact with body fluids, dead bodies, or infectious fomites. Rigorous attention to appropriate infection control practices has proven to be effective in interrupting transmission. Filovirus outbreaks are associated with high case-fatality rates, generally ranging from 25% to 90% (*16*). Exposure to imported animals and subsequent person-to-person spread caused a cluster of Marburg hemorrhagic fever cases in several European cities in 1967 (*13*). Although filovirus infection is primarily limited to sporadic outbreaks in the African continent, recent infections of tourists from Europe and the United States have been documented (*13*,*17*). Despite the low incidence of filovirus infections, their occurrence in outbreaks with high rates of death and the potential to spread to places away from the original focus has prompted investments in enhancing surveillance mechanisms and focused research in developing effective therapies and vaccines. Basic research on filoviruses is limited by the need to conduct the work in high-containment laboratories.

### Anthrax

In the United States, naturally occurring anthrax infection is more commonly reported in animals than in humans (*18*,*19*). Naturally occurring anthrax cases have been associated with direct contact with infected animals, occupational exposure during industrial processing of infected animal products, and production or use of drums made from contaminated hides. Anthrax cases resulting from these forms of exposure are very rare in much of the industrialized world because of improvements in hygiene and use of modern animal husbandry practices and reduced use of contaminated imported raw materials in industrial processing of animal products (*19*,*20*). Inhalation anthrax is the rarest of the 3 common forms of anthrax (cutaneous, gastrointestinal, and inhalation), but it has the highest case-fatality rate. During 2009–2010, anthrax among persons injecting heroin was reported primarily in Scotland but also in other European countries, adding a new route of infection (*20*). Spore formation, persistence in the environment, ease of dissemination, inhalation route of transmission, and associated high rates of death make *Bacillis anthracis* one of the most serious bioterrorism agents. The 2001 anthrax outbreak in the United States from the mailing of spore-laden envelopes highlighted the need for preparedness and countermeasure efforts to mitigate the effects of intentionally released *B. anthracis* (*21*).

## Role of Infectious Disease Pathology

Unexplained sudden illnesses and deaths can be sentinels for the recognition of newly emerging infections and for the early detection of outbreaks of naturally occurring or intentionally released infectious agents. If laboratory tests are negative or inconclusive and a patient dies, thorough pathologic investigation aides in identifying the etiologic agent. Over the past several decades, the Centers for Disease Control and Prevention (CDC) has effectively used infectious disease pathology to diagnose the causes of sudden illness and death and to assist in identifying sources of multiple high-profile outbreaks, many of which were caused by new and reemerging etiologic agents.

When hantavirus pulmonary syndrome was first identified in 1993 in the Four Corners area of the southwestern United States, pathologic examination was critical for characterizing the illness and contributed to discovery of the etiologic agent (*22*). Autopsy and examination of biopsied tissues played a major role in the investigation of the bioterrorism-related anthrax cases in 2001 in the United States. During the early phase of the epidemic of respiratory syndrome (SARS) in 2003, investigations focused on characterizing the etiologic agent of what appeared to be a severe respiratory illness spreading among household contacts and to health care workers. Attempts to identify an infectious agent by standard laboratory testing failed to produce consistent results. As the number of SARS-related deaths increased, specimens examined by virus isolation techniques, electron microscopy, and pathologic examination led to identification of the causative agent of SARS as a novel coronavirus (*23*). More recently, infectious disease pathology has been instrumental in the investigation of a multistate outbreak of fungal meningitis associated with epidural injection of steroid preparations and in the identification of several organ transplant–associated infections, such as lymphocytic choriomeningitis virus, *Balamuthia* disease, and rabies.

## Conclusions

We briefly described only selected examples of low-incidence, high-consequence pathogens. Many other similar infectious diseases with relatively low incidence but high-rates of death occur in many parts of the world. Besides those mentioned above, other low-incidence, high-consequence pathogens described in this issue of Emerging Infectious Diseases include Crimean-Congo hemorrhagic fever and Rift Valley fever. Ongoing surveillance and public health research of high-consequence pathogens are critical for identifying their natural reservoirs, developing diagnostic tests, and devising appropriate control and prevention measures. Studying the molecular characteristics of these pathogens is critical to understanding their pathogenesis and ultimately to developing vaccines and antimicrobial drugs. Despite low incidence of these diseases, maintaining a preparedness posture to tackle the challenges posed by the emergence or reemergence of some of these pathogens should remain a priority. Public health resources are wisely spent by adequately preparing for the inevitable emergence or reemergence of infectious diseases that might currently be of low incidence but have the potential to spread to immunologically naive populations. The application of the age-old tools of pathology bolstered with a wide array of bioassays, developed by using modern advances in molecular diagnostics, has helped CDC tackle old infectious disease challenges and newly emerging and reemerging diseases. Advanced molecular detection approaches in concert with infectious disease pathology can play a prominent role in emergency preparedness and in addressing the public health challenges of the future.
